# Effects of ITO Contact Sizes on Performance of Blue Light MicroLEDs

**DOI:** 10.1186/s11671-022-03754-9

**Published:** 2022-11-28

**Authors:** Yu-Hsuan Hsu, Yu-Yun Lo, Yi-Hsin Lin, Hsiao-Wen Zan, Ray-Hua Horng

**Affiliations:** 1 Department of Photonics, College of Electrical and Computer Engineering, National Yang Ming Chiao Tung University, Hsinchu, 30010 Taiwan, ROC; 2 Institute of Electronics, National Yang Ming Chiao Tung University, Hsinchu, 30010 Taiwan, ROC

**Keywords:** Micro-LED, Contact ratio, Blue light, Current crowding

## Abstract

In this study, the effect of ITO contact ratio for blue light micro-light-emitting diode (µLED) with dimensions 40 μm × 40 μm was assessed. The contact ratio from 0.2 to 0.8 was designed for the ratio of electrode area to light-emitting area. As the contact ratio increased from 0.2 to 0.8, the turn-on voltage of µLED decreased. It could be due to the short lateral diffusion length in multiple quantum wells (MQW) and lower parallel resistance for the µLED with a large contact ratio. The leakage currents of single µLED were below 5.1 × 10^–9^ A, no matter the contact ratio. It means that the contact ratio does not affect the leakage current as measured on single chip. Moreover, µLED array with a 0.8 contact ratio presented the highest output power than other samples (5.25 mW as the current density of 1875 A/cm^2^). It could attribute to the MQWs usage, the metal contact reflective behavior and less current crowding, which generated more carriers and extracted more lighting from the µLED. The simulation data using SpeCLED software agreed well with these experiments, and µLED with a 0.8 contact ratio showed the best optoelectronic properties.

## Introduction

The leading display technologies are based on liquid–crystal display (LCD) and organic light-emitting diode (OLED), which almost dominate the display market. Even though LEDs as the backlight for LCD are very mature, due to high-efficiency, brightness, color purity, and self-emission [1, 2], micro-light-emitting diodes (µLEDs) have attracted much attention in high-resolution display applications. Compared with traditional LCDs, µLEDs exhibit higher energy efficiency, resolution, contrast, stability, and longer life. They can also be applied to very huge, transparent, or wearable displays of any shape [3, 4]. Moreover, developing smartphones, pads, wearable devices, and augmented/virtual reality (AR/VR) products lead to a growing commercial demand for high-performance µLEDs. However, a full-color µLEDs display on the same active materials, including red, blue, and green subpixels, is the primary requirement for simplified processing. Traditionally, InGaN epitaxy layers were used for blue and green color LEDs, and AlGaInP materials were applied to fabricate red color LEDs [5–7]. Integrating full-color subpixels is challenging due to the heterostructure and different substrates. To solve this problem, different colors of quantum dots (QDs) excited by a high-energy light source, such as UV and blue-light wavelengths, were developed in the past years [8–10].

Although LEDs have many advantages in optoelectronic performances, as the size of the chip shrinks to below 100 µm (called µLEDs), some issues were induced and affected the properties of devices significantly [11, 12]. For example, sidewall defects are created by an inductively coupled plasma reactive ion etching (ICP-RIE) [13–15]. These defects result in the Shockley–Read–Hall non-radiative recombination and further decreasing the efficiency of µLEDs. Previous studies were focused on the smooth morphology of the sidewall and passivation by a high-quality passivation layer using atomic layer deposit [16–18], which can repair the sidewall defect and avoid the non-radiative recombination behavior. Nevertheless, the electrode dimension effects on the performance of µLEDs were not studied, especially for the high-efficiency blue light µLEDs. To prevent deviations from the fabricated process of µLEDs, a chip size of 40 μm × 40 μm was used in this study. Meanwhile, the effect of the contact ratio can be observed more clearly in this chip size. The contact ratio of the ITO and electrode area to the emission area from 0.2 to 0.8 was studied. There were top-electrodes of µLEDs with 8 μm × 8 μm, 16 μm × 16 μm, 24 μm × 24 μm, and 32 μm × 32 μm, respectively, for the 0.2, 0.4, 0.6, and 0.8 contact ratios. The optoelectronic characteristics of µLEDs were measured and evaluated, including emission output power, wall-plug efficiency (WPE), and external quantum efficiency (EQE). We also discuss herein a simulation of the current distribution and characteristics for different contact ratio chips.

### Experimental

**Fig. 1:**
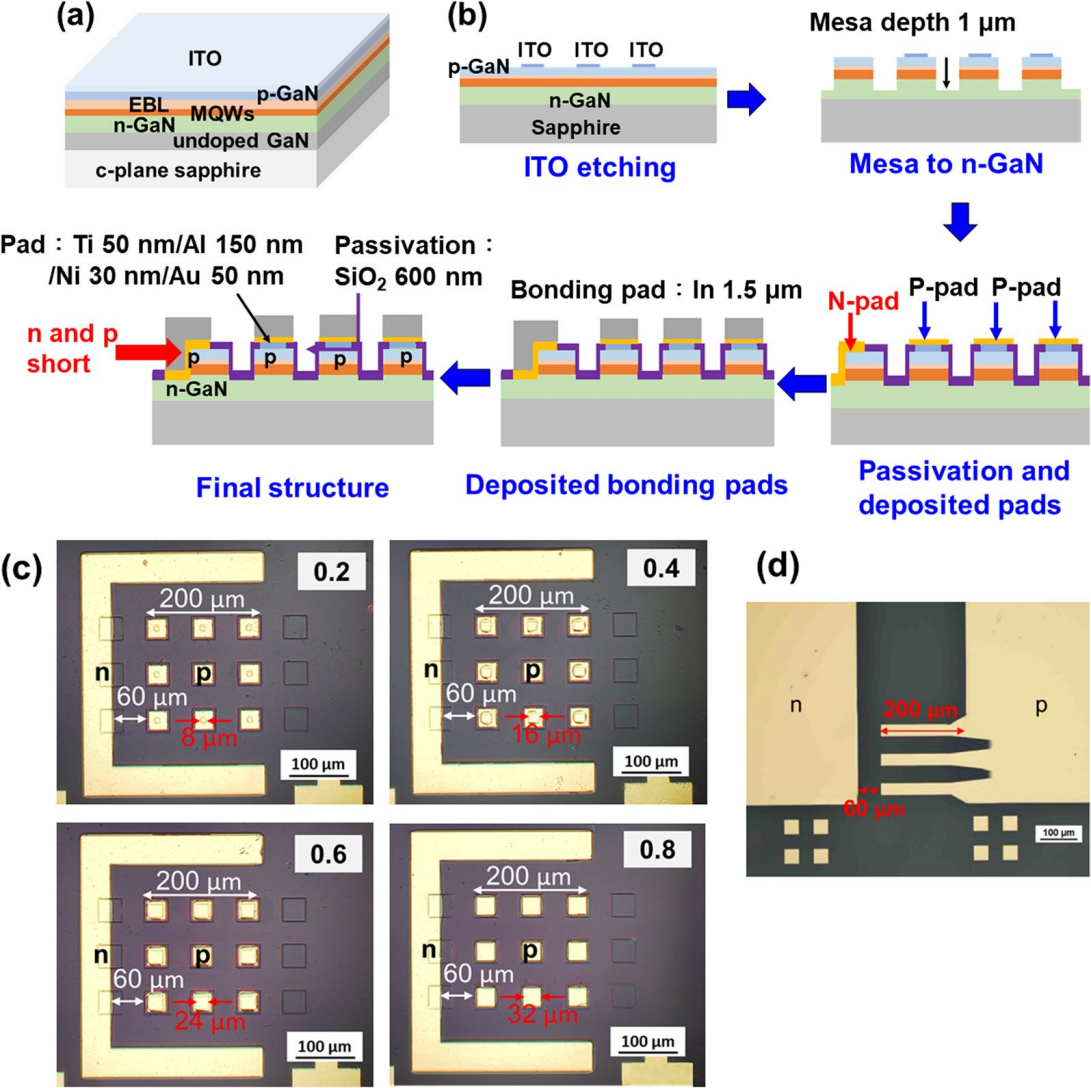
**a** epi-layer structure, **b** μLED fabricated process flow, **c** 3 × 3 arrays, and **d** the circuit design in this study

The µLEDs were grown on sapphire substrate with 450 nm wavelength blue light emission. First, a buffer GaN was grown on c-plane sapphire, then grown a Si-doped n-GaN layer. The Mg-doped p-GaN layer and the active layer were contained by InGaN/GaN multiple quantum wells (MQWs). Therefore, an electron blocking layer was stacked between p-GaN and MQWs, which could control the current flow direction and prevent overflow leakage from the n-GaN layer to the p-GaN layer. A 300 nm ITO was grown on the p-GaN epilayer for the top Ohmic contact processing. Figure 1a showed the epilayer structure of blue light LEDs, and the schematic of µLED fabrication in this study was also presented shown in Fig. 1b. The standard LED development processes, such as photolithography, ICP-RIE dry etching, dielectric passivation layer deposited using plasma-enhanced chemical vapor deposition (PECVD), metal evaporation, and lift-off for the contact pads, are shown in Fig. 1b. First, the indium tin oxide (ITO) layer was wet-etched using an ITO etching solution and defined the four-size contact sizes on the µLEDs with 40 μm × 40 μm dimension. The mesa structure was obtained by GaN etching with the gas of 20 sccm Cl_2_ and 30 sccm BCl_3_ in 5 mTorr to determine the active region 40 × 40 μm by ICP-RIE and the etching depth was 1 μm. After that, the 600 nm SiO_2_ passivation layer was grown by PECVD, and the dry etching process opened for the metal contact area. Meanwhile, the n and p electrodes of the stack of Ti/Al/Ni/Au with 50 nm/150 nm/30 nm/50 nm were deposited on the passivation layer with open contact pads by an E-gun evaporation system, then deposited 1.5 μm thick indium with 32 μm × 32 μm by a thermal evaporator for the flip-chip process. Moreover, in order to let the bonding pads for n and p have the same height, the n ohmic contact pad was deposited on the sidewall and connected to p layer of one chip, shown in the red arrow in Fig. 1b. On the other hand, it is necessary to bond the μLED to the circuit board using the similar flip chip technology. If there is no sidewall protection using the passivation layer, the μLEDs should be easily short or present large leakage after bonding to circuit board.

To investigate the effect of different metal contact areas in μLED device, a laser direct writing system (MLA-150, Heidelberg Instruments) was applied for the photolithography process without the mask in this study, which technology can align accurately for smaller pattern design and also can reduce the cost to fabricate the mask. The size of μLED chips was 40 μm × 40 μm, and the ITO contact sizes were adjusted from 8 μm × 8 μm to 32 μm × 32 μm, as shown in Fig. 1c. The output power of single µLEDs was too low to measure. Therefore, the µLEDs array with three by three was designed. The metal layers of Ti/Al/Ni/Au (50/500/30/60 nm) deposited on the double-sides polished sapphire substrate were used as circuit electrodes for the µLEDs array package (Fig. 1d). Finally, the µLEDs array was flip chip bonded on the circuit under a bonding temperature of 220 °C and the pressure of 25 N for 5 min. After packaged, the light emission of the µLEDs array was from the backside of sapphire.

After processing, the electrical and optical properties of all µLEDs were measured at room temperature. Current–voltage (*I*–*V*) characteristics of the single chip µLED were investigated using a semiconductor parameter analyzer (2400 Source Meter; Keithley) by on-wafer measurement. Then, a charge-coupled device (CCD) was used to observe the corresponding emission pattern. After the In-Au bonding and TO-Can packaging, the output powers of the μLED array were measured with a calibrated integrating sphere.

Besides μLED manufacture, the results of the simulation by SpeCLED were also presented. Based on the power device parameters (power chip size in 1000 μm × 1000 μm at the operation current 350 mA), the total input current at 0.56 mA (current density 35 A/cm^2^) was used for the µLEDs with 40 μm × 40 μm dimension. The properties of the ITO contact ratio of µLEDs with contact ratios from 0.2 to 0.8 were evaluated and discussed by current distribution, light output power, external quantum efficiency (EQE), and wall-plug efficiency (WPE).

## Result and discussion

**Fig. 2:**
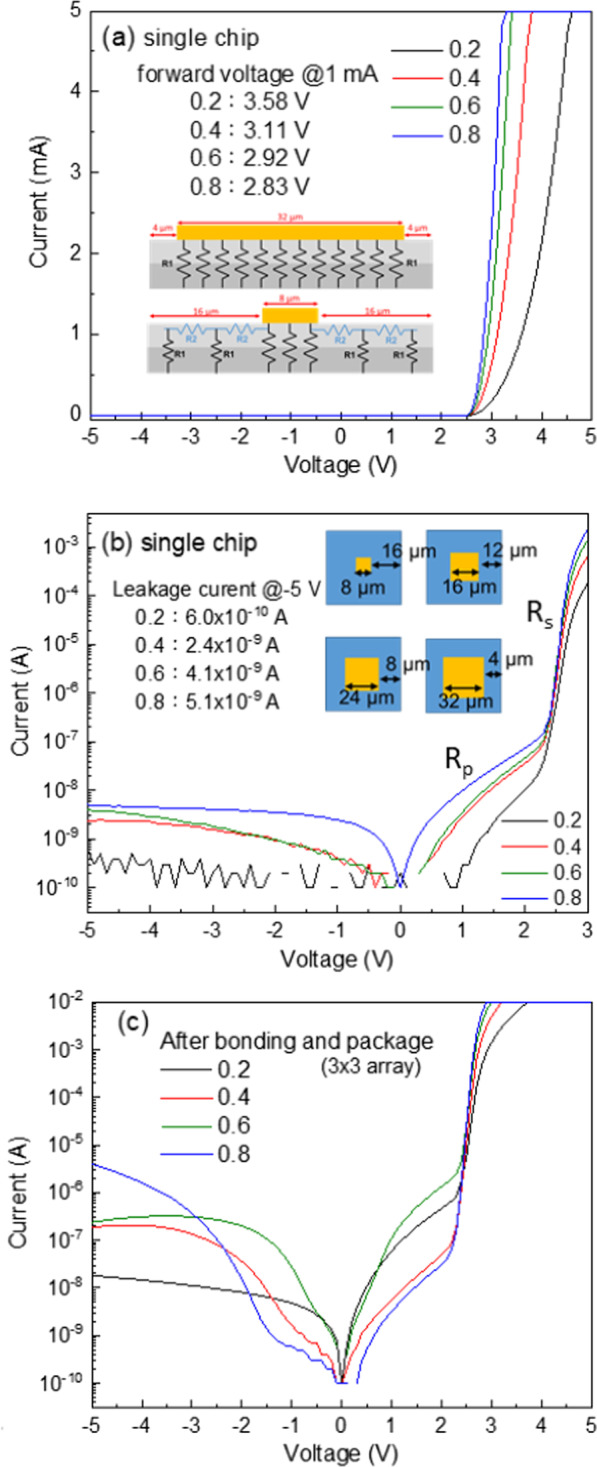
I-V characteristic **a** in linear-scale **b** log scale on-chip measurement, and **c** I–V characteristic in log scale after flip-chip and wire bonding from − 5 to 5 V of different contact ratios

Figure 2A shows the forward I-V characteristics of the single chip µLED with the different contact ratio. The turn-on voltage of single chip µLED with the contact ratio from 0.2 to 0.8 was 2.79, 2.61, 2.57, and 2.54 V, respectively. The corresponding dynamic resistance was 16.67, 4.62, 2.17 and 1.3 kΩ at the voltage of 3 V. When the ITO contact ratio increased from 0.2 to 0.8, the forward voltage of µLEDs array decreased from 3.58 to 2.83 V at 1 mA injection current. The µLEDs with contact ratios of 0.6 and 0.8 presented lower turn-on voltage and dynamic resistance. It could be attributed to the larger ITO contact pad area resulting in the lower current density at the same current injection. As the µLED was turned on, there existed many parallel resistances (R1), as shown in the inset of Fig. 2a. It can contribute to low equivalent resistance, which can be fitted and calculated in Fig. 2b. The parallel resistances R1, which can be calculated as the I-V operated at the low voltage range shown in Fig. 2b, were obtained at 1.16, 0.94, 0.93, and 0.63Ω when the contact ratio varied from 0.2 to 0.8. For a 0.2 contact ratio, the ITO contact pad of the µLED was only 8 μm × 8 μm, and for a 0.8 contact ratio, the dimension of the ITO contact pad of the µLED was 32 μm × 32 μm. Besides the parallel resistances under the pad, there is an additional equivalent series resistance R2, as shown in the inset of Fig. 2a. This series resistance decides how current laterally spread out of the contact area and flows into QW. It can be calculated as the I–V operated at the high voltage range shown in Fig. 2b. Hence, the total resistance was not directly inversely proportional to the contact pad area. Because of lower equivalent resistance, a lower forward voltage was observed for the µLED with contact ratios of 0.6 and 0.8 samples under the same injection current. A large contact pad area can contribute to the uniform current distribution, resulting in low resistance. As the contact ratio increased from 0.2 to 0.8, the value of series resistances R2 could decrease and was calculated at 250.6, 173.4, 120.4, and 117.4Ω, respectively.

The main issue in µLED with a large contact pad is the leakage current, which affects device properties significantly. Figure 2b shows the reverse current as a function of reverse voltage for all µLEDs. As the contact ratio increased, the leakage currents were slightly raised from 6 × 10^–10^ A to 5.1 × 10^–9^ A measured from single chip before bonding. The leakage current of µLEDs with different contact ratios was all less than 5.1 × 10^–9^ A at the reversed voltage of − 5 V. Although the distance from the edge of the ITO contact pad to the sidewall was only 4 μm for µLED with a contact ratio of 0.8, as shown in the inset of Fig. 2b, the leakage current was still very low. This indicated that in this chip size, the leakage current did not increase as the contact ratio increased, and a weak relationship was observed between the leakage behavior and the contact pad area. The first reason would be that the SiO_2_ passivation layer deposited by PECVD can effectively protect the sidewall area from leakage for µLED with a 0.8 contact ratio with 4 µm distance from the edge of ITO to side wall. The other reason would be the relatively more considerable sheet resistance in the p-GaN layer (due to the low mobility hole), which limits the spread of the current. The lower lateral carrier diffusion length (< 5 µm) in the InGaN quantum well was due to the random alloy fluctuation [19, 20]. Nevertheless, the SiO_2_ passivation could be contribute to the low leakage current because the metal contact pad and ITO to the sidewall was only 4 μm for µLED with a contact ratio of 0.8 which is within the hole diffusion length. The above I-V characteristics exhibited that the contact ratio affects the resistance, which further affects the forward voltage in each µLED. Therefore, the ideality factor of 0.2, 0.4, 0.6, and 0.8 contact ratios were calculated using the slopes of the I-V curve in the log scale and also listed in Table 1. The ideality factor exhibited the highest value in 0.2 contact ratio in 2.43. As the contact ratio increased from 0.4, 0.6 to 0.8, the corresponding ideality factor of µLED decreased in 2.19, 2.02, and 1.95. It was well known that the recombination carrier of the current domain is in a small forward voltage, the ideality factor is generally 2.0. Exceeding 2.0 is due to the defect-assisted tunneling domain of the conducting mechanism [19, 21]. In this result, the contact ratio of 0.6 and 0.8 present better electrical properties. As concerning the µLEDs with 0.2 and 0.4 contact ratio, due to higher resistance and low current spreading, defect-assisted tunneling could be easily occurred in these two samples.

**Table 1:** Contact size and electric-optical properties in this study

40 × 40 μm	Contact size	Ideality factor	Output power_max_	WPE_max_ (%)	EQE_max_ (%)
0.2	8 × 8 μm	2.43	2.27 mW	9.61	9.43
0.4	16 × 16 μm	2.19	3.51 mW	10.46	10.16
0.6	24 × 24 μm	2.02	4.63 mW	11.08	10.76
0.8	32 × 32 μm	1.95	5.25 mW	11.24	10.84

Furthermore, the leakage current of µLEDs was also measured after the flip-chip. The current in the log scale as a function of voltage is presented in Fig. 2c. The leakage current of the nine µLEDs at -5 V increased from 1.8210^–8^ to 3.9510^–6^ A as contact ratio increased from 0.2 to 0.8. Obviously, after flip chip packaged, the larger contact resulted in the leakage current increasing even the chips has been passivated by SiO_2_. Therefore, it is important to evaluate whether the contact ratio affects the optoelectronics in the µLEDs with 3 × 3 arrays.

**Fig. 3:**
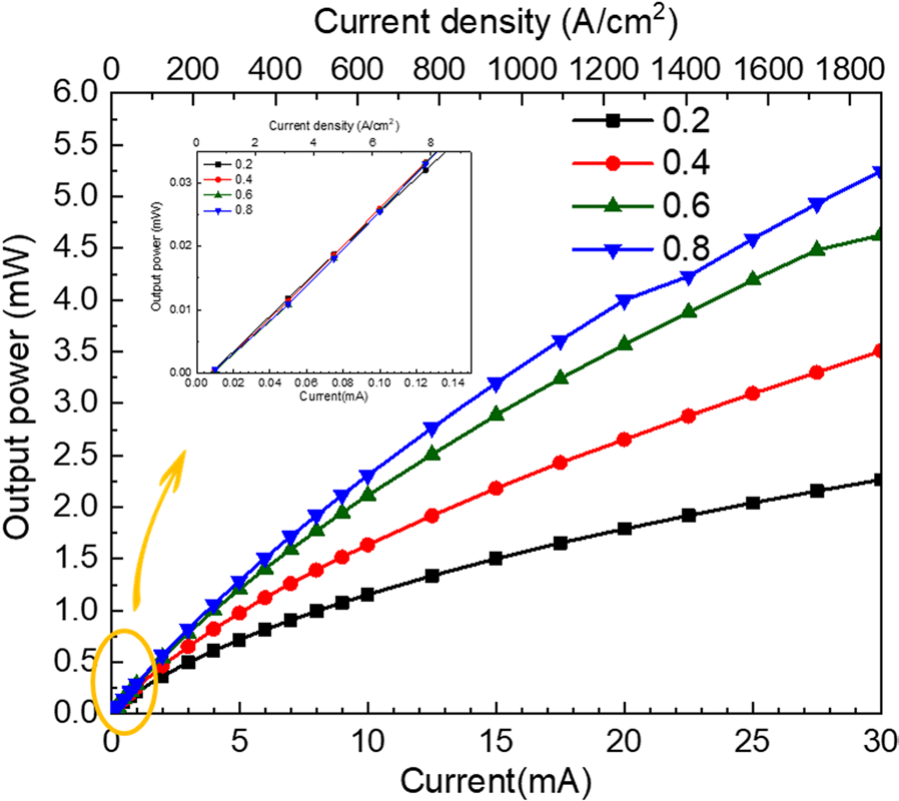
The emission output power of different contact ratios

Figure 3 shows the optical output power of the nine µLEDs array as a function of the injection current and injection current density (top x-axial). With the increase in the injection current, the output power increased for all µLEDs array with different contact ratios, and the µLEDs array with 0.8 contact ratio had a larger slope than the other µLEDs. When the injection current increased from 0.01 to 30 mA, the output powers of µLEDs array with 0.2, 0.4, 0.6, and 0.8 contact ratios were raised from 0.0006 to 2.27 mW, 0.004 to 3.51 mW, 0.004 to 4.63 mW, and 0.004 to 5.25 mW, respectively. The inset of Fig. 3 showed the emission output power in a small injection current. The output power of the 0.2 contact ratio was slightly higher than other contact ratios when the injection currents were 0.05 and 0.075 mA. The 0.2 contact ratio has a higher local current density at the same total current; therefore, it reached the internal quantum efficiency (IQE) peak earlier than other cases, leading to the highest output power. However, the droop effect also happens earlier as the current density increases, which lead to a smaller output power curve slope. The highest output power of µLEDs array was in contact ratio 0.8 as injection current from 0.1 to 30 mA. If the contact ratio is too small, the current crowding affected the performance obviously, which also has a poor spread of the current in a small contact ratio. The electrical characteristic of the µLEDs array also affected the emission output power. Moreover, the µLEDs with a larger contact ratio can contribute to more light reflecting toward the sapphire side and enhance the output power [22]. In this work, the performance of µLEDs were evaluated by injection current from 0.01 to 30 mA. Because the injected current was used to drive 9 chips, it means 1 µA to 3.33 mA for one chip with 40 µm 40 µm. This discussion current is a good range which cover high IQE at low current density for VR application and high power output at high current density for AR application.

**Fig. 4:**
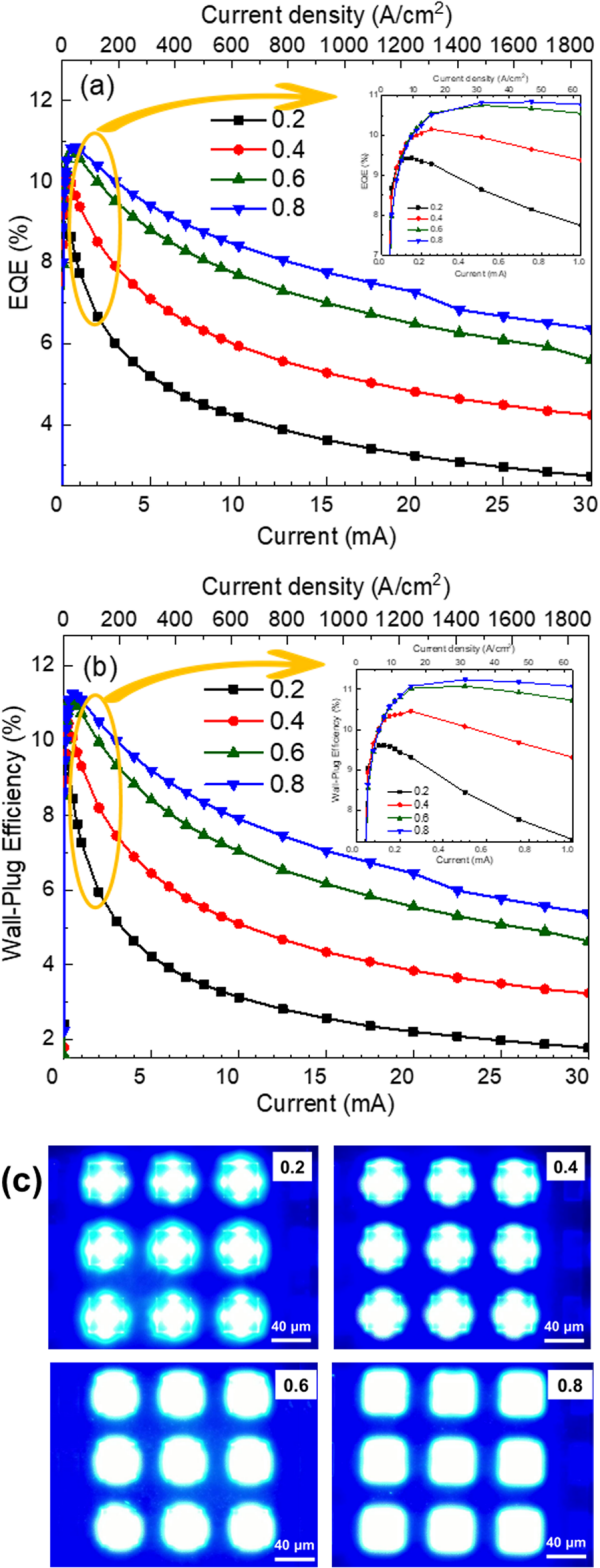
**a** EQE, **b** wall-plug efficiency of different contact ratios, and **c** emission pictures at 1 mA (62.5 A/cm^2^) for different contact ratio

The EQE as the function of injection current and injection current density was plotted and is shown in Fig. 4a. In the small injection current region as shown in the inset of Fig. 4a, the µLED array with small contact ratio 0.2 presented the highest EQE than those of µLEDs with other contact ratios as injection small current at 0.05 and 0.1 mA. However, as the injection current increased from 0.05 to 30 mA, the EQE curve of µLED array with a 0.2 contact ratio dropped significantly as compared to those of µLED array with the wide contact ratios in Fig. 4. This phenomenon was also observed in the emission output power shown in Fig. 3. The µLED with 0.2 contact ratio has outstanding performance than other µLEDs in small injection current because the higher current density was obtained for the µLEDs with a 0.2 contact ratio. Nevertheless, as the injection current increased, the current crowding occurred and the current spreading limited the usage of MQWs, which caused the EQE to decrease intensely from 9.43 to 2.72% of µLED with 0.2 contact ratio. In contrast, the EQE of µLEDs array with 0.8 contact ratio decreasing was alleviated from 10.84 to 6.35% as the injection current increased to 30 mA. The highest EQE value was observed at injection currents 0.15, 0.25, 0.5, and 0.75 mA as the contact ratio increased from 0.2 to 0.8, and the EQE were 9.43, 10.16, 10.76, and 10.84%, respectively.

The other important property is the WPE of these devices. The WPE as a function of current injection for the µLED with different contact ratios is presented in Fig. 4b. As the injection current increased, the WPE of µLEDs array with contact ratios 0.2, 0.4, 0.6, and 0.8 decreased from 9.61 to 1.79%, 10.46 to 3.24%, 11.08 to 4.63%, and 11.24 to 5.38%, respectively. The inset of Fig. 4b shows the WPE of µLEDs array at a small current injected from 0.05 to 1 mA. The obtained results were very similar to those of EQE results discussed above. In the µLEDs array with 0.2 contact ratio, the WPE of µLEDs array was slightly higher than other contact ratios at 0.05 mA injection current, then decreased dramatically when the injection current increased from 0.1 to 30 mA; it could be because of a higher forward voltage in the 0.2 contact ratio. Since the WPE is output light power/IV, the applied voltage was much larger in the case of a 0.2 contact ratio. The µLEDs with a 0.8 contact ratio presented the highest WPE at 11.24%. The above conclusion can be also demonstrated by the emission patterns for the µLEDs with different contact ratios as 1 mA injection current, as shown in Fig. 4c. The emission area presented the largest and most uniform for the µLEDs with a 0.8 contact ratio. At a contact ratio below 0.4, the light is only emitted around the contact area. Due to the less usage of the MQW area, the carrier reconvened only in the injection region. Meanwhile, the emission uniformity at the uLEDs with 0.2 and 0.4 contact ratios were both very disappointing and emitting an asymmetric pattern. In contrast, the uLED with 0.6 and 0.8 contact ratios presented more symmetry and uniformity. Furthermore, the uLEDs with 0.8 contact ratio presented a square shape which was matched to the injection area than that of uLED with 0.6 contact ratio.

**Fig. 5:**
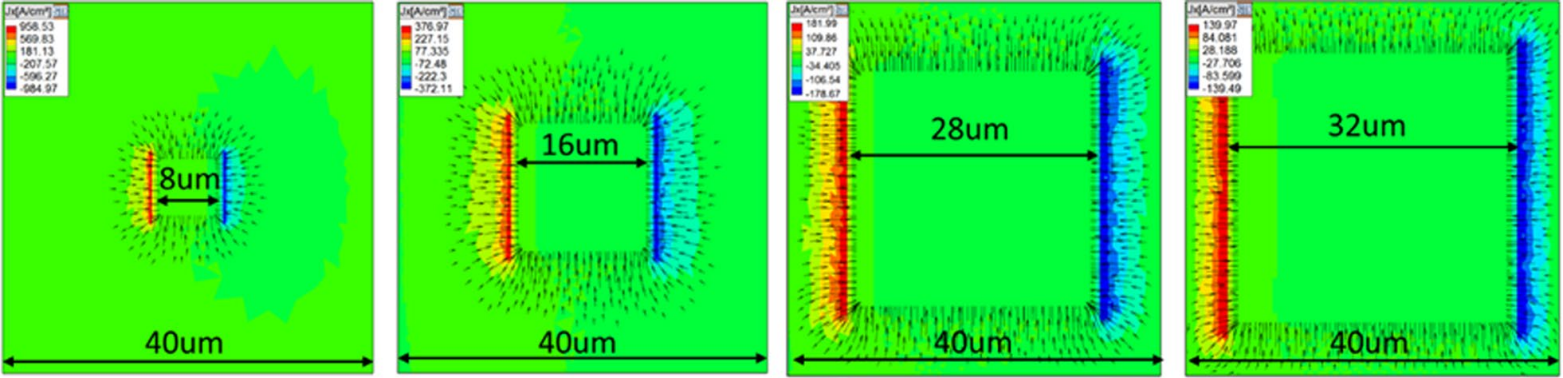
Current spreading simulation results for different contact ratios

Finally, to understand the lateral carrier distribution, the SpeCLED software was used to simulate the current density distribution for µLED with different ITO contact ratios. Figure 5 presents the simulation results of current density distribution in the lateral direction for µLEDs with different contact ratios. A constant injection current, 0.56 mA, was used to drive the µLEDs for the simulation. The obtained characteristics of the device calculated by simulation software, including the forward voltage, output power, wall-plug efficiency, and EQE, are listed in Table 2. This table shows that as the contact ratio increased from 0.2 to 0.8, forward voltage decreased from 2.80 to 2.59 V. This simulation result was consistent with the measurement data from the experiment performed in this study. As mentioned above, the parallel resistance decreased as the contact ratio increased, which led to a decrease in the forward voltage. Moreover, emission output power, wall-plug efficiency, and EQE were all enhanced as the contact ratio increased. The uLEDs with contact ratio 0.8 exhibited the highest output power, wall-plug efficiency, and EQE peak. The values were 0.204 mW, 14.04%, and 11.73%, respectively. The obtained simulation results agreed well with the experiment results.

**Table 2:** Simulation data of different contact ratios in 40 μm × 40 μm chip size

40 μm × 40 μm	0.2	0.4	0.6	0.8
Forward bias [V]	2.80	2.65	2.61	2.59
Current [mA]	0.56			
Output power [mW]	0.125	0.164	0.187	0.204
Wall-plug efficiency [%]	7.98	11.12	13.02	14.04
EQE [%]	7.21	9.52	10.97	11.73

## Conclusion

IN this study, the 3 × 3 blue light µLEDs arrays were fabricated for four ITO contact ratios in 40 × 40 μm^2^ with contact sizes 8 × 8, 16 × 16, 24 × 24, and 32 × 32 μm^2^ using a laser direct writing system without a mask. The turn-on voltage decreased from 2.8 to 2.5 V as the contact ratio increased due to the lower parallel resistance in widely contact ratio. Moreover, the leakage currents were below 5.1 × 10^–9^ A at the reverse bias − 5 V. The obtained results suggest that the passivation layer plays an excellent role in repairing the sidewall defect in these chip sizes and also indicate that the contact size did not induce leakage current in the bottom emitting µLEDs. Particularly, the contact ratio of 0.2 presented a higher emission output power and EQE in the small injection current region. This is attributed to the higher current density in the small injection current than the wide contact ratios. However, it further increased the injection current, the current crowding, and the current spreading will domain the optoelectronic performances in µLEDs. The optoelectronic characteristics exhibited an outstanding performance of the µLEDs contact ratio was 0.8. When injected with 0.5 mA current (current density 31.3 A/cm^2^), the 0.8 contact ratio had the highest wall-plug efficiency and EQE; the values were 11.24% and 10.84%, respectively. Among this contact ratio, 0.8 also presented the strongest output power, 5.25 mW, as the injection current was 30 mA (current density 1875 A/cm^2^).

The same result was obtained from the simulation; when the contact ratio increased, the turn-on voltage decreased from 2.8 to 2.6 V. The contact ratio of 0.8 had more outstanding properties than others. When the contact ratio increased from 0.2 to 0.6, the value of output power, wall-plug efficiency, and EQE increased significantly. Meanwhile, according to the current distribution, the MQWs usage efficiency was observed distinctly. It resulted that the 0.8 contact ratio showed an outstanding performance in this study.

## Data Availability

The datasets used and/or analyzed during the current study are available from the corresponding author on reasonable request.

## Abbreviations

µLEDs: Micro-light-emitting diodes
ICP: Inductively coupled plasma
PECVD: Plasma-enhanced chemical vapor deposition
WPE: Wall-plug efficiency
EQE: External quantum efficiency
LCD: Liquid–crystal display
OLED: Organic light-emitting diode
AR/VR: Augmented/virtual reality
QDs: Quantum dots
ALD: Atomic layer deposit
MQWs: Multiple quantum wells
EBL: Electron blocking layer
